# Spatial distribution of arable and abandoned land across former Soviet Union countries

**DOI:** 10.1038/sdata.2018.56

**Published:** 2018-04-03

**Authors:** Myroslava Lesiv, Dmitry Schepaschenko, Elena Moltchanova, Rostyslav Bun, Martina Dürauer, Alexander V. Prishchepov, Florian Schierhorn, Stephan Estel, Tobias Kuemmerle, Camilo Alcántara, Natalia Kussul, Maria Shchepashchenko, Olga Kutovaya, Olga Martynenko, Viktor Karminov, Anatoly Shvidenko, Petr Havlik, Florian Kraxner, Linda See, Steffen Fritz

**Affiliations:** 1 International Institute for Applied Systems Analysis, Laxenburg A-2361, Austria; 2 Bauman Moscow State Technical University, Mytischi 141005, Russia; 3 University of Canterbury, Christchurch 8140, New Zealand; 4 Lviv Polytechnic National University, Lviv 79013, Ukraine; 5 University of Dąbrowa Górnicza, Dąbrowa Górnicza 42300, Poland; 6 Department of Geosciences and Natural Resource Management, University of Copenhagen, Øster Voldgade 10, København K, 1350, Denmark; 7 Institute of Environmental Sciences, Kazan Federal University, Kazan, Tovarishcheskaya str.5, Kazan 420097, Russia; 8 Leibniz Institute of Agricultural Development in Central and Eastern Europe (IAMO), Department of Structural Development of Farms and Rural Areas, Theodor-Lieser-Straße 2, D-06120 Halle, Germany; 9 Department of Earth & Environment, Boston University, 685 Commonwealth Avenue, Boston, MA 02215, USA; 10 Geography Department, Humboldt-University Berlin, Unter den Linden 6, 10099 Berlin, Germany; 11 Integrative Research Institute for Transformations in Human-Environment Systems (IRI THESys), Humboldt-University Berlin, Unter den Linden 6, 10099 Berlin, Germany; 12 Departamento de Geomática e Hidráulica, División de Ingenierías, Universidad de Guanajuato, Av. Juárez 77, Zona Centro, Guanajuato 36000, Gto. Mexico; 13 Space Research Institute NAS Ukraine and SSA Ukraine, 40 Glushkov Ave 03680, Kiev 187, Ukraine; 14 Russian Institute of Continuous Education in Forestry, Pushkino 141200, Russia; 15 Dokuchaev Soil Science Institute, Moscow 109017, Russia

**Keywords:** Climate sciences, Ecology, Agriculture, Environmental chemistry, Environmental social sciences

## Abstract

Knowledge of the spatial distribution of agricultural abandonment following the collapse of the Soviet Union is highly uncertain. To help improve this situation, we have developed a new map of arable and abandoned land for 2010 at a 10 arc-second resolution. We have fused together existing land cover and land use maps at different temporal and spatial scales for the former Soviet Union (fSU) using a training data set collected from visual interpretation of very high resolution (VHR) imagery. We have also collected an independent validation data set to assess the map accuracy. The overall accuracies of the map by region and country, i.e. Caucasus, Belarus, Kazakhstan, Republic of Moldova, Russian Federation and Ukraine, are 90±2%, 84±2%, 92±1%, 78±3%, 95±1%, 83±2%, respectively. This new product can be used for numerous applications including the modelling of biogeochemical cycles, land-use modelling, the assessment of trade-offs between ecosystem services and land-use potentials (e.g., agricultural production), among others.

## Background & Summary

After the Soviet Union collapsed, abandonment of agricultural land in the former Soviet Union (fSU) countries occurred as a result of the restructuring of the economy and the adjustment towards open-market conditions from 1990 to 2010 (refs 1–3). These major land-use changes have had a significant impact both regionally and globally, e.g. Schiernhorn *et al*.^4^, which illustrates impacts beyond the borders of the fSU.

Unfortunately, we still have limited knowledge of the spatial distribution of abandoned land in the fSU countries. Accurate spatial information on land abandonment is required for many studies, e.g. as a benchmark for monitoring cropland expansion and highlighting areas suitable for biomass production, but also to pinpoint opportunities for increasing ecosystem services, such as carbon sequestration on abandoned lands and increasing habitats for umbrella species^5–9^. However, existing global land cover/land use maps suffer from a high level of uncertainty e.g. refs 10–12 and are not tailored towards the identification of abandoned land. For example, the global land cover time series from 1992 to 2015, produced in the framework of the Climate Change Initiative (CCI) of the European Space Agency (ESA)^13^, do not account for any losses in cropland over this time period yet the area sown shrank by 42.5 Mha between 1990-2010 according to national Russian statistics^14^. Usually global mapping initiatives, such as the ESA CCI, focus on certain types of land cover change to satisfy the needs of one group of users, addressing the needs of other users only partially. For the development of this recent ESA CCI land cover product, the CCI community did not prioritize mapping of cropland change but rather focused on forest loss and gain.

At the same time there have been efforts to map abandoned land of small study plots as well as regionally^15–18^. For example, Prischepov et al.^15^ have developed a map of abandoned arable land at a 30 m resolution for a few study plots in Russia, Poland and Lithuania, covering the period 1990–2000 while Kraemer et al.^17^ have mapped a cropland time series for 1990–2010 based on Landsat imagery covering two study plots in Kazakhstan. Another example is a map of farmland abandonment by Estel et al.^18^, which is based on MODIS time series that covers all of Europe for the period 2001–2012. The spatial and temporal extent of these maps is different, as well as the definitions for abandoned arable land, which makes it impossible to compare these maps directly. Moreover, these maps do not fully cover Kazakhstan or the non-European part of Russia. Hence there is a clear need to develop an accurate map of abandoned land that covers the whole fSU.

This paper presents a state of the art hybrid map of current arable and abandoned land for eight fSU countries (Armenia, Azerbaijan, Belarus, Georgia, Kazakhstan, Republic of Moldova, Russian Federation and Ukraine). By fusing the best available, global and regional spatial information together, the map provides information on land abandonment by 2010. We have used training data in the data fusion methodology, which were collected by visual interpretation of very high resolution (VHR) imagery using Geo-Wiki^19,20^, to increase the quality of the map. With a second independent Geo-Wiki data set, we have assessed the accuracy of this product.

## Methods

In this study, we aimed to collect and fuse existing sources of information, including indicators of land abandonement derived from remote sensing data. These include abandoned arable land maps that were produced by classification of Landsat imagery^17^; classification of MODIS-based time series of Normalized Difference Vegetation Index (NDVI)^18,21^; or downscaling of statistical data on abandoned land based on the calculation of a “so-called” cropland suitability index^1^. Among different existing data fusion approaches, e.g. regression, decision trees or neural networks, we have chosen the Naïve Bayes classifier^22^. Naïve Bayes is the basic form of a Bayesian Network and, as such, is a direct implementation of the Bayes’ theorem. It is easy to implement, can be updated dynamically, and deals easily with missing data. Moreover, it has been shown to perform well on most classification tasks and is often significantly better than other classification methods^23,24^.

**Figure 1:**
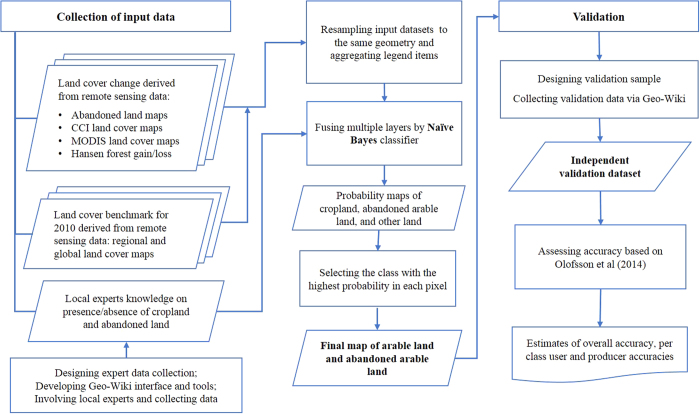
A flowchart of the methodology used to create the hybrid map of arable and abandoned land.

Figure 1 presents a flowchart of the methodology used to create the hybrid map of arable and abandoned land. We first collected land cover maps from different epochs as well as regional maps of abandoned land. Moreover, with the help of regional experts using the Geo-Wiki^19,20^ land cover tool, we developed a reference (training) data set on arable and abandoned land, using visual interpretation of VHR historical imagery from Google and Bing. We then integrated the different land cover and abandoned land maps with the Geo-Wiki reference data set using a data fusion algorithm to produce a hybrid map of arable and abandoned land. The target resolution of the final product is 10 arc-second (ca 300 m at the equator) to match the geometry and spatial resolution of two input products: the hybrid global land cover map^25^ and the ESA CCI land cover 13 products.

### Map legend and definitions

As one of the inputs, we used land cover maps that include cropland as a land cover class. However, cropland or arable land is a land use class according to the definition provided by the Food and Agriculture Organization (FAO) of the United Nations. Therefore, in this paper, we refer to arable and abandoned as land use classes.

National statistics on land include the following land use classes based on definitions from FAO^26^ with specific regional differences:

- ***Arable land*** is land under temporary crops, temporary meadows for mowing or pasture, land under market or kitchen gardens and land temporarily laid fallow (less than five years). Temporarily fallowed land is land set aside for one or more years before being cultivated again.
- ***Sown area*** refers to the area on which sowing or planting has been carried out for the crop under consideration on the soil prepared for that purpose. (http://faostat.fao.org/site/375/default.aspx).
- ***Fallow land*** (temporary) is the cultivated land that is not sown for one or more growing seasons. The maximum idle period is usually less than five years. Land remaining fallow for too long may acquire characteristics requiring reclassification, such as "permanent meadows and pastures" (if used for grazing), "forest or wooded land" (if overgrown with trees), or "other land" (if it becomes wasteland).
- ***Agricultural land*** refers to the land area that is arable, under permanent crops, or under permanent pastures and hayfields.

The hybrid map developed here consists of three land use classes: arable land in use, abandoned arable land, and other land uses (e.g. urban, forest, etc.).

1. Arable land includes sown area and bare fallow (cultivated, but not seeded)
2. Abandoned arable land is the land that was previously cultivated (i.e. belongs to the agricultural land use class) but has not been utilized for more than 5 years^1,27,28^. “Abandoned arable land” is almost never reported, and is calculated as the difference between the total arable land and the utilized arable land.
3. Other land use is the land that is currently not and has never been utilized for agricultural purposes or it was formerly arable land that is now occupied by infrastructure so it can no longer be considered as potentially available for agricultural purposes.

### Input maps

To be used as input data, we collected maps that provide us with the following information:

1. Abandonment of arable land derived from remote sensing data, such as abandoned land from Alcantara^21^, abandoned land from Prishchepov^15^, etc.
2. Series of annual land cover maps, such as MODIS land cover^29^ and CCI land cover^13^. These maps provide additional information on the transition of land cover from one type to another, e.g. from cropland to grassland, shrubland or forest.
3. Land cover maps and cropland maps for 2010. There are many more land cover maps available for the year 2010 than for earlier years. Some maps for 2010 are more accurate than the maps for 2000 or older because it is possible to obtain better training data for the most recent years, e.g. GlobeLand30^30^ for 2010 compared to 2000. We consider these maps useful for delineating active cropland for 2010 and other land cover classes that are mapped with high accuracies, e.g. water, forest and bare land.

Table 1 lists the land cover and land use maps that we used as inputs to produce the hybrid map and the correspondence to the land use classes of arable utilized land, arable abandoned land, and other land uses. We then resampled the input data sets to the target resolution of 10 arc-second. In the first step we simplified the legends by merging some of the land cover classes that are similar but not relevant to agriculture, e.g. different types of forest (Supplementary Table S1). We then aggregated those maps at a lower resolution than 10 arc-seconds to a 10 arc-second resolution: for categorical data, we applied a majority rule while for continuous data, we calculated the mean. We then resampled all maps to the same grid by applying the nearest neighbor technique. Finally, we converted continuous variables (e.g. percentage cropland) to categorical ones by using a 50% threshold. Table 1 also shows the spatial and temporal coverage of each input data set.

### Geo-Wiki reference data on abandoned land

**Figure 2:**
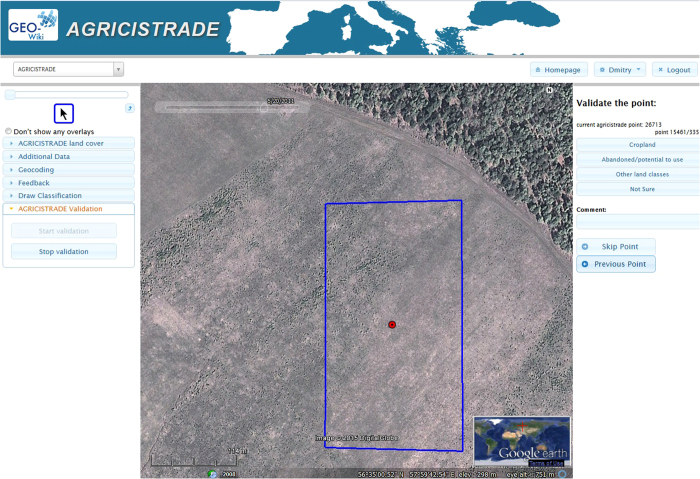
Screenshot of the Geo-Wiki interface to collect expert training data.

We collected reference data on abandoned land through the Geo-Wiki platform (http://geo-wiki.org), which allows users to classify Google Earth and Bing VHR imagery. An example of the interface is provided in Figure 2. The blue box corresponds to a 10-sec pixel; in the top left corner is a time slider to view available historical imagery at this location while the user chooses the classes from the right hand panel.

Twenty experts from the IIASA Geo-Wiki network along with partners from the AGRICISTRADE project took part in an imagery classification campaign; together they collected information at ca 15K points. These expert data were then used for training a Bayesian network to fuse the input data sets into a hybrid product.

**Figure 3:**
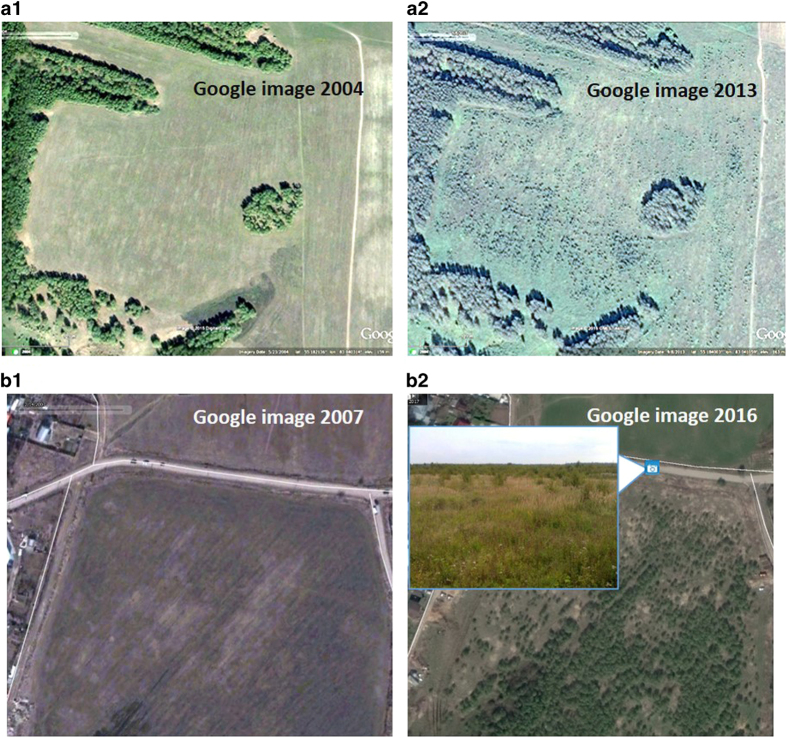
Examples (Geo-Wiki screenshots) of abandoned land. (a1) Coordinates 55.18 N 83.04 E. The image from 2004 shows cropland. (a2) Coordinates 55.18 N 83.04 E. The image from 2013 is abandoned land. (b1) Coordinates 56.02 N 37.88 E. The image from 2007 shows cropland. (b2) Coordinates 56.02 N 37.88 E. The image from 2016 and the ground truth photo from 2015 confirms that it is now abandoned land.

As part of the data collection process, we asked the experts to determine if each pixel had greater than 50% arable land, 50% abandoned arable land or 50% other land. When it was impossible to define a unique class, the experts had the option to choose “Not Sure” (see Figure 2). We excluded “Not sure” locations in training the Bayesian network. The experts examined both historical imagery at each location and historical profiles of NDVI. Figure 3 provides an example of how historical VHR imagery in Geo-Wiki was used to identify abandoned land in two different cases. In particular, the increased number of shrubs over time, which is clearly visible in Figure 3, is a visual sign of abandonment. Abandoned land may include not only abandoned arable land but also abandoned pastures.

### Bayesian network

We combined the input data sets with the Geo-Wiki reference data set using a Bayesian network to produce a hybrid map of arable and abandoned land. The Naïve Bayes classifier has been shown to perform well in classification problems e.g. refs 38,39. One of the advantages of this method is the ease with which it incorporates input data sources that have differing classifications. This means that there is no need to translate land cover classes into the same legend, e.g. the forest gain map by Hansen can indicate areas where forest gains have taken place on formerly cultivated agricultural lands^39^. In addition, some of the input data sets provide information for only part of the fSU region e.g. 1,15,21 but the Naïve Bayes classifier can handle missing data. Finally, this approach allows us to use input data with different temporal extents. We considered the Geo-Wiki reference data set as the truth.

We have applied the Naïve Bayes classifier as follows. Let *G*_*i*_ be the truth in location *i*, and $\left\{S\right\}=\{S_{1i},S_{2i},\ldots ,S_{ki}\}$ be the readings of the *k* satellites in that location. In general, one can partition the set of satellite observations (input maps) into conditionally independent subsets: $\left\{S\right\}=\left\{\left\{S^{\left(1\right)}\right\},\left\{S^{\left(2\right)}\right\},\ldots ,\left\{S^{\left(J\right)}\right\}\right\},$ where J≤K is the number of such subsets. The Bayes’ formula used is:

(1)Pr(G|{S})=Pr({S}|G)Pr({S})=∏jPr({S(j)}|G)Pr(G)∑g∏jPr({S(j)}|G=g)Pr(G=g)

We estimated the conditional probabilities $\mathrm{Pr}(\{S^{\left(j\right)}\}|G)$ from the contingency tables for the classifications obtained through Geo-Wiki and the k^th^ input map classification. The region-specific prior probabilities Pr(G) were assumed to be equal.

If the data are only available for a subset {S*} of {S} and missing for the rest, denoted here as $\left\{\bar{S}\right\}$, then the probability becomes:

(2)Pr(G|{S*})=∑S¯∏SPr(S|G)Pr(G)∑g∑S¯∏SPr(S|G=g)Pr(G=g)=∏S*Pr(S|G)Pr(G)∑g∏S*Pr(S|G=g)Pr(G=g)

because $\sum^{\;}\mathrm{Pr}\left(S|G\right)=1.$ Thus, if no information is available from a given input data source, the corresponding terms are simply omitted from the model.

Usually after abandonment, agricultural land transforms into another land cover class, either grassland, shrubland or forest. This transformation depends on human impact, bioclimatic zone, altitude, and other factors. Therefore, the Naïve Bayes classifier was run at the ecozone level^40^ in order to delineate different transformation processes that follow after land is abandoned. For example, abandoned croplands in forested regions in Ukraine and Belarus will be afforested over years, while abandoned croplands in the steppe regions of Siberia and in Kazakhstan will revert to grasslands. Note that we initially ran a series of tests with different strata, such as the whole study region or with national boundaries. However, this resulted in massive ovestimation of abandoned land and was therefore abandoned in favour of the ecozone stratification.

From the application of the Bayesian approach, we obtained a probability map of cropland, abandoned arable land, and other land (summing to 1 in each pixel). Then we selected the class with the highest probability in each pixel to produce the final hybrid map product.

### Example of applying the Naïve Bayes classifier at the pixel level

The following provides an example of how the Naïve Bayes classifier operates at the pixel level using a a simple situation where observations of only two satellites S_A_ and S_B_ are available. The satellite S_A_ classifies observations into 3 classes, A_1_, A_2_, and A_3_, whereas the satellite S_B_ classifies observations into 2 classes, B_1_ and B_2_. The conditional probabilities of observing each of the classes in arable land, or abandoned arable, or other land are given in Table 2 and Table 3, respectively G_1_, G_2_, G_3_. Thus, for example, the satellite S_A_ will assign the abandoned land to classes A_1_, A_2_, and A_3_ with probabilities 0.8, 0.2, and 0.0 respectively, and these probabilities will sum to one. These probabilities are calculated from the Geo-Wiki reference data on abandoned land.

Suppose now, that we want to estimate the probability that a cell assigned to classes A_1_ and B_2_ by satellites S_A_ and S_B_ respectively, is arable. Assuming that the prior probabilities of each class (G_1_, G_2_, G_3_) are equal to 1/3 (we rounded it to 0.3), then:

$$\begin{array}{l} \mathrm{Pr}\left(G1|A1,B2\right)=\\ =\frac{\mathrm{Pr}\left(G1|C1\right)\mathrm{Pr}\left(B2|G1\right)\mathrm{Pr}\left(G1\right)}{\mathrm{Pr}\left(A1|G1\right)\mathrm{Pr}\left(B2|G1\right)\mathrm{Pr}\left(G1\right)+\mathrm{Pr}\left(A1|G2\right)\mathrm{Pr}\left(B2|G2\right)\mathrm{Pr}\left(C2\right)+\mathrm{Pr}\left(A1|G3\right)\mathrm{Pr}\left(B2|G3\right)\mathrm{Pr}\left(G3\right)}\\ =\frac{0.8*0.4*0.3}{0.8*0.4*0.3+0.1*0.8*0.3+0.1*0.5*0.3}=0.86, \end{array}$$

Note that the classes for the two satellites do not need to be in any way compatible, nor do they need to correlate strongly with the variable of interest G In terms of the estimator performance. The best results are achieved when, for any source and class C, Pr(C|G_1_) differs substantially from Pr(C|G_2_) or Pr(C|G_3_). On the other hand, one can see that when Pr(C|G_1_)=Pr(S|G_2_)= Pr(C|G_3_) for any class, the posterior distribution Pr(G_1_|$\left\{S^{*}\right\}$) will always equal the prior distribution Pr(G_1_). Thus, the observations will be completely uninformative.

### Recommendation for mapping abandoned land in other regions of the world

The methodology presented here could be used for mapping abandoned land in other regions of the world. Two components are needed: (i) the input maps of land cover, cropland and abandoned arable land (if available) corresponding to the regions of interest; and (ii) the reference data set on abandoned arable land. The latter data set can be collected from field data or from very high resolution satellite data using an application such as Geo-Wiki or Collect Earth (http://www.openforis.org/tools/collect-earth.html). The spatial resolution of the map produced using the methodology outlined here should be dependent on the size of the abandoned fields.

## Data Records

The two data records are provided in zipped files (.zip):

- a 10 arc-second raster in GeoTiff format (the legend is presented in Table 4) (Data Citation 3);
- the validation data set as a .csv table with 5782 records (see Table 5 for the data set structure) (Data Citation 4).

**Figure 4:**
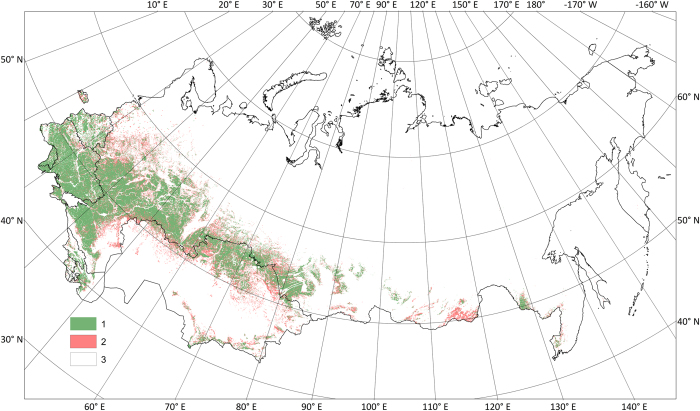
Spatial distribution of arable and abandoned land in the fSU. Legend items: 1- arable land, 2-abandoned land, 3-other land.

Figure 4 shows the hybrid map of arable and abandoned land in the fSU countries, presented in this paper.

The map is also available from the Geo-Wiki Agricistrade page, where we overlaid it on top of Google Maps and Bing satellite imagery using Open Layers. Users can examine the map by zooming into specific locations or gain an overview of the map by panning around the region.

## Technical Validation

We have validated the hybrid map by following the procedure set out in Olofsson *et al*.^41^, which allows for the estimation of confidence intervals and adjusted areas based on confusion matrices. The validation sample design follows a two-step random stratified approach:

1. The first stratum is by country/region: Russia, Belarus, Moldova, Kazakhstan and Ukraine as individual countries and Armenia, Azerbaijan and Georgia grouped together as the “Caucasus” region;
2. The second stratum is by mapped class: arable utilized, abandoned land and other land cover types.

The final sample consists of 5972 pixels at a 10 arc-second resolution by country/region as follows: 1504 sample pixels in Russia; 911 in Belarus; 923 in Moldova; 915 in Kazakhstan; 922 in Ukraine; and 797 in the Caucasus. We randomly distributed the pixels across the countries with an increased number of samples in rare classes, i.e. utilized arable and abandoned land. We invited regional experts from Ukraine and Russia to classify the sample by visual interpretation of VHR historical imagery available from Google and Bing in Geo-Wiki. The experts were asked to identify the dominant land use in each sample pixel, i.e. arable utilized, abandoned land or other land. If it was difficult to determine a unique class, the experts were asked to select one of the following classes: “not sure if arable or abandoned land”, “not sure if arable or others”, “not sure if abandoned land or other land”. These “not sure” sample sites were used in the accuracy assessment. For example, if a validation site was classified as “not sure if arable utilized or abandoned land” and the mapped class was arable, then a value of 0.5 was added to the cell of the confusion matrix in the row mapped class “arable” and column reference class “arable” while the other 0.5 was added to the cell in the row mapped class “arable” and column reference class “abandoned land” (Table 6).

There are many challenges in mapping abandoned land, which are difficult to tackle and which result in low user accuracies for this land use class, for example:

- In Moldova and Caucasus, the fields are much smaller than a 10 arc-second grid, and there are many orchards that are confused with abandoned land from remote sensing;
- In the forest-steppe and forest zones of Ukraine and Belarus, where the majority of abandoned lands are allocated in these countries, the landscapes are very fragmented and therefore difficult to map from remote sensing;
- In Kazakhstan, abandoned lands change from arable to grassland, which is the land cover transition type that is very difficult to map in the steppe zone with a very dry climate;Table 7
- In Russia, due to its large territory, there are abandoned lands in the forest zone with high fragmentation, and there are abandoned fields in the steppe.

**Figure 5:**
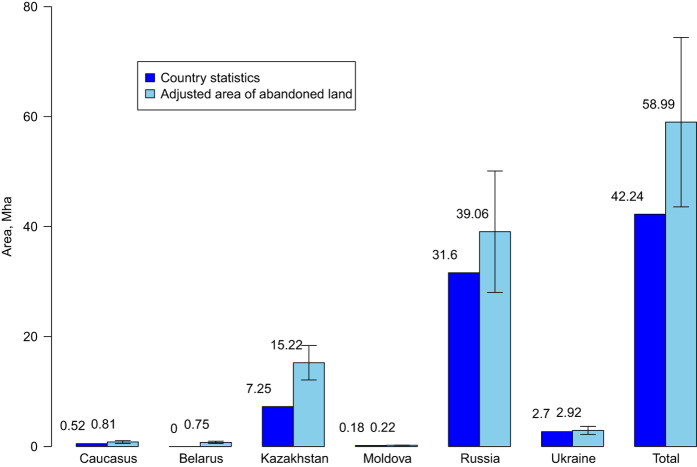
Area estimates for abandoned land.

Figure 5 presents the area estimates for abandoned land (95% confidence interval). We calculated the country statistics based on official country reports as the difference between the arable and cultivated area^42–49^. The adjusted areas were calculated based on the confusion matrices (Supplementary Table S2, Supplementary Table S3, Supplementary Table S4, Supplementary Table S5, Supplementary Table S6, Supplementary Table S7) by following the procedure set out in Olofsson *et al*.^41^ In Figure 5, for Kazakhstan, the error bar from the map is not within the official estimates so it indicates underestimation by the official statistics. The overall error bar is also outside the total abandoned land area, indicating that the overall abandoned land area in the fSU is underestimated by the statistics. In comparing the estimates across the fSU countries, the widespread underestimation of abandoned land in the official national statistics due to deliberate manipulation for administrative reasons e.g. ref. 50 should be considered.

In addition to the accuracy assessment presented above, we compared the hybrid map produced here with the latest ESA CCI land cover maps^51^ covering the period 1992–2012. To undertake this comparison, we first generated a derivative ESA CCI product containing information on cropland gain and loss over the period 1992–2012. From this derivative product, the cropland loss and gain for fSU countries was estimated to be approximately 2.3 and 5.4%, respectively. Thus the overall trend based on ESA CCI is cropland expansion (especially in Kazakhstan) rather than an increase in the area of abandoned land. This is contrary to what has been published in all other studies^1,17,21^ and according to the official statistics reported by each country.

## Usage Notes

The hybrid map reported in this paper represents a novel arable and abandoned land product, which covers more than 90% of all agricultural lands across the fSU, and has many potential uses. For example, the map can be used for assessment of the biogeochemical cycles (e.g., carbon dynamic) on abandoned and cultivated fields^1,8,52,53^, for the analysis of the patterns and proximate causes of greening (vegetation recovery) and browning (vegetation degradation)^54–57^, for investigation into the drivers of land abandonment and the implications for ecosystem services and biodiversity. The product can be used at the original resolution (10 arc-second with pixel size of approximate 4–7 ha) or aggregated to a coarser resolution such as 1 to 10 km. We envision a good alignment with and improvement of global land-use data sets such as HYDE 3.1 (ref. 58), KK11 (ref. 59), and the SAGE Global Land-Use Database^60^.

The hybrid map can serve as an input to a regional or country level analysis since we have achieved reasonable accuracies.

## Additional information

**How to cite this article**: Lesiv, M. *et al.* Spatial distribution of arable and abandoned land across former Soviet Union countries. *Sci. Data* 5:180056 doi: 10.1038/sdata.2018.56 (2018).

**Publisher’s note:** Springer Nature remains neutral with regard to jurisdictional claims in published maps and institutional affiliations.

## Supplementary Material



Supplementary Information

## Figures and Tables

**Table 1:** Land use classes and coverage of the input data sets.

Data set	Mapped classes	Spatial and temporal coverage		
	Arable utilized land	Abandoned land	Other land	
MODIS land cover^29^	√	√^a^	√	Global, 2001-2010
CCI land cover^13^	√	-	√	Global, 2000, 2010
IIASA-IFPRI cropland^12^	√	-	√	Global, 2005
GLC-SHARE^31^	√	-	√	Global, 2014
GlobeLand30^30^	√	√^b^	√	Global, 2000, 2010
Abandoned land from Schierhorn^1^	√	√	√	European Russia, Ukraine
Abandoned land from Prishchepov^15^ (Data Citation 1)	√	√	√	fragments of European Russia and Belarus
Areas sown from de Beurs^16^	√	-	-	fragment of European Russia
Russian land cover^32^	√	√	√	Russia, 2009
Forest cover from Hansen^33^	√^c^	-	√	Global, 2010
Land cover map from Alcantara^21^	√	√	√	Belarus, Moldova, European Russia 2009
Cropland from Kraemer^17^ (Data Citation 2)	√	√	√	Northern Kazakhstan
Abandoned from Estel^18^	√	√		Belarus, Moldova, Ukraine, European Russia 2010
Cropland from Bartalev^34^	√	-	√	Russia, 2012
Cropland from Kussul^35,36^	√	-	√	Ukraine, 2010
√data set contains corresponding class.				

^a^proxy for abandoned land, which was estimated based on the area that MODIS land cover classified as cropland in 2001 and was then changed to a different land cover class, i.e. not cropland in 2010, even though we recognize that this product was not designed for change analysis.

^b^arable land abundance estimated as the difference between the amount of arable land between 2000 and 2010.

^c^dense forest cover excluding cropland.

**Table 2:** Satellite A: Conditional Probabilities of observing classes A_1_, A_2_, and A_3_ for arable land (G_1_), abandoned arable(G_2_), and other land(G_3_) respectively.

Classes	A_1_	A_2_	A_3_
G_1_	0.8	0.2	0.0
G_2_	0.1	0.6	0.3
G_3_	0.1	0.3	0.6

**Table 3:** Satellite B: Conditional Probabilities of observing classes B_1_ and B_2_ for arable land (G_1_), abandoned arable(G_2_), and other land(G_3_) respectively.

Classes	B_1_	B_2_
G_1_	0.6	0.4
G_2_	0.2	0.8
G_3_	0.5	0.5

**Table 4:** Legend of the hybrid map.

Raster value	Class
1	Arable land
2	Abandoned land
3	Other land

**Table 5:** Validation data set structure.

Field	Description
Id	Unique id
Lat	Latitude
Lon	Longitude
Class_id	Land use class:1 – arable land2 - abandoned land3 - other land12 - can be either arable or abandoned land13 - can be either arable or other land23 - can be either abandoned land or other land
Class_name	Class names that correspond to the Class_id above

**Table 6:** Example of counting for “not sure” validation points in confusion matrices.

Map/Validation dataset	Arable land	Abandoned land	Other land
**(a)**			
Arable land	0.5	0.5	
Abandoned land			
Other land			
**(b)**			
Arable land	0	1	
Abandoned land			
Other land			
Mapped class is “arable”: (a) a validation pixel identified as “not sure if arable utilised or abandoned land”; (b) a validation pixel identified as “abandoned land”.			

**Table 7:** Accuracy measures for the hybrid map.

Accuracy indicators	Countries						
	Caucasus	Belarus	Kazakhstan	Moldova	Russia	Ukraine	
Overall accuracy %:	90±2	84±2	92±1	78±3	95±1	83±2	
Arable:	User accuracies,%	68±7	86±4	86±6	81±3	86±4	86±3
	Producer accuracies,%	86±5	88±3	78±6	94±1	78±6	97±1
Abandoned land:	User accuracies,%	33±9	22±7	46±9	18±8	33±7	31±9
	Producer accuracies,%	55±16	65±15	76±11	35±14	47±14	62±13
Other land:	User accuracies,%	98±1	95±2	98±1	91±4	98±1	96±3
	Producer accuracies,%	91±1	82±2	95±1	49±4	97	64±3
These presents the results of the accuracy assessment. Supplementary Tables S2 to S7 contain the confusion matrices for individual countries and the Caucasus region.							
