# Supplementary material for: Spatial distribution of arable and abandoned land across former Soviet Union countries
Source: Sci Data. 2018 Apr 3;5:180056. doi: 10.1038/sdata.2018.56 (PMC5881411; doi:10.1038/sdata.2018.56)
Supplement: Supplementary Information [file sdata201856-s2.pdf]

Supplementary materials

List of Tables:

Table S1**:** Aggregation of land cover classes for the input maps 2
Table S2: Confusion matrix for Russia 5
Table S3: Confusion matrix for Ukraine 6
Table S4: Confusion matrix for Kazakhstan 7
Table S5: Confusion matrix for Moldova 8
Table S6: Confusion matrix for Belarus 9
Table S7: Confusion matrix for the Caucasus 10

References…….. 11

Table S1: Aggregation of land cover classes for the input maps

Dataset	Original classes	Aggregated class
MODIS land cover[1]	Evergreen needleleaf forest, Evergreen broadleaf, Deciduous needleleaf, Deciduous broadleaf, Mixed forest	Forest
	Closed shrublands, Open shrublands	Shrubland
	Woody savannas	Woody savannas
	Savannas	Savannas
	Grasslands	Grasslands
	Permanent wetlands	Wetland
	Croplands	Croplands
	Cropland/Natural vegetation mosaic	Cropland/Natural vegetation mosaic
	Cropland in 2001 converted to non-cropland in 2010	Abandoned cropland
	Cropland mosaic in 2001 converted to non-cropland in 2010	Abandoned cropland mosaic
	Barren or sparsely vegetated	Barren
	Urban and built-up	Urban
	Water	Water
	Snow and ice	Snow and ice
CCI land cover[2]	Cropland rainfed, Cropland irrigated,	Cropland
	Mosaic cropland (>50%) / natural vegetation, Mosaic natural vegetation (>50%) / cropland,	Mosaic natural/cropland
	Grassland, Herbaceous cover, Mosaic herbaceous cover (>50%) / tree and shrub	Grassland
	Shrubland, Tree or shrub cover	Shrubland
	Tree cover broadleaved evergreen, broadleaved deciduous, needleleaved evergreen, needleleaved deciduous,	Tree cover
	Sparse vegetation (tree, shrub, herbaceous cover), Sparse shrub (<15%), Sparse herbaceous cover (<15%)	Sparse vegetation
	Shrub or herbaceous cover flooded	Wetland
	Urban areas	Urban

	Permanent snow and ice, Lichens and mosses	Snow and ice
	Bare areas	Bare
	Water	Water
Cropland IIASA[3], GLC share[4]	No cropland	No cropland
	1-20 % cropland	Minor cropland
	21-60 % cropland	Major cropland
	61-100 % cropland	Dominant cropland
GlobLand30[5]	Mosaic cultivated 20-50 %	Mosaic cultivated
	Cultivated in 2000 converted to other classes	Abandoned
Cropland by Kraemer[8]	Arable	Arable
	Abandoned	Abandoned
	Other	Other
Sown by de Beurs[10]	Arable	Arable
Abandoned by Prishchepov[7], Cropland by Alcantara[9]	Managed agricultural land	Managed agricultural land
	Abandoned	Abandoned
	Other	Other
Abandoned by Schierhorn[6]	Cropland	Cropland
	Abandoned	Abandoned
	Other	Other
Russian land cover[11]	Pine, Spruce, Larch, Cedar, Birch, Oak, Other tree species	Forest
	Sparse forest	Sparse forest
	Burnt	Burnt
	wetland	wetland
	cropland	cropland
	hayfield/pasture	hayfield/pasture
	abandoned arable	abandoned arable
	grassland	grassland
	shrubland	shrubland
	water	water
	unproductive	unproductive
Forest by Hansen[12]	No tree cover	Treeless
	1-40 % tree cover	Sparse tree cover
	41-80 % tree cover	Open tree cover
	81-100 % tree cover	Close tree cover
Abandoned by Estel[13]	Not arable	Other
	2001-2006 cultivated, 2007-2012 - abandoned	5-6 years abandoned
	4 years abandoned out of 12	4 years abandoned
	3 years abandoned out of 12	3 years abandoned
	Not cultivated 2001-2012	Permanently abandoned
Cropland by Bartalev[14]	Cropland	Cropland
	Other	Other
Cropland by Kussul[15,16]	Artificial, Bare Land	Artificial/ bare
	Crops	Crops
	Forest	Forest

	Grassland	Grassland
	Water	Water
	Urban	Urban
	Aggregated pixel contains 20-60% of cropland	cropland 20-60%
	Cropland mosaic in 2000 converted to non-cropland in 2010	abandoned 20-60 %
	Cropland 2000 converted to non-cropland in 2010	abandoned 60-100%

Accuracy estimates by countries/regions

All the calculations were done in R, version 3.4.0. R using package "dtwSat", which has functions based on the algorithms found in Olofsson et al.[17,18]

Table S2: Confusion matrix for Russia

Land use classes	Mapped area, Mha	Weights	Map/ Validation data set	1	2	3	Sum	User accuracies	Confidence interval
1	119.02	0.07	1	284	7.5	39.5	331	0.86	0.04
2	56.21	0.03	2	37.5	52.5	70	160	0.33	0.07
3	1,512.40	0.90	3	10.5	12	990.5	1013	0.98	0.01
Total	1,687.63	1	Sum	332	72	1100	1504		
			Producer accuracies	0.78	0.47	0.97			
			Confidence interval	0.06	0.14	0.00			

Overall accuracy:	95%
Confidence interval:	1%
Confidence level	95%

Adjusted area, Mha +/-

Land use class	Adjusted area, Mha	Error, Mha
1	130.97	11.08
2	39.06	11.05
3	1517.6	14.99

Table S3: Confusion matrix for Ukraine

Land use classes	Mapped area, Mha	Weights	Map/ Validation data set	1	2	3	Sum	User accuracies	Confidence interval
1	39.99	0.67	1	501	12.5	67.5	581	0.86	0.03
2	5.58	0.09	2	18.5	36	60.5	115	0.31	0.09
3	14.26	0.24	3	5	4	217	226	0.96	0.03
Total	59.83		Sum	524.5	52.5	345	922		
			Producer accuracies	0.97	0.62	0.64			
			Confidence interval	0.01	0.13	0.03			

Overall accuracy: 83%
Confidence interval: 2%
Confidence level 95%

Adjusted area

Land use class	Adjusted area, Mha	Error, Mha
1	35.73	1.22
2	2.92	0.73
3	21.38	1.23

Table S4: Confusion matrix for Kazakhstan

Land use classes	Mapped area, M ha	Weights	Map/ Validation data set	1	2	3	Sum	User accuracies	Confidence interval
1	28.69	0.10	1	121.5	3.5	16	141	0.86	0.06
2	25.06	0.09	2	24.5	52.5	37	114	0.46	0.09
3	230.35	0.81	3	5	8.5	646.5	660	0.98	0.01
Total	284.10		Sum	151	64.5	699.5	915		
			Producer accuracies	0.78	0.76	0.95			
			Confidence interval	0.06	0.11	0.01			

Overall accuracy:	92%
Confidence interval:	1%
Confidence level	95%

Adjusted area

Land use class	Adjusted area, Mha	Error, Mha
1	31.85	2.94
2	15.22	3.13
3	237.03	3.63

Table S5: Confusion matrix for Moldova

Land use classes	Mapped area, Mha	Weights	Map/ Validation data set	1	2	3	Sum	User accuracies	Confidence interval
1	2.60	0.77	1	446.5	114	92.5	653	0.81	0.03
2	0.22	0.07	2	39	18.5	42.5	100	0.18	0.08
3	0.55	0.16	3	11.5	3	155.5	170	0.91	0.04
Total	3.38		Sum	497	135.5	290.5	923		
			Producer accuracies	0.94	0.35	0.49			
			Confidence interval	0.01	0.14	0.04			

Overall accuracy:	78%
Confidence interval:	3%
Confidence level	95%

Adjusted area:

Land use class	Adjusted area, Mha	Error, Mha
1	2.60	0.09
2	0.22	0.04
3	0.55	0.09

Table S6: Confusion matrix for Belarus

Land use classes	Mapped area, Mha	Weights	Map/ Validation data set	1	2	3	Sum	User accuracies	Confidence interval
1	8.20	0.40	1	297	4.5	42.5	344	0.86	0.04
2	2.25	0.11	2	37	29.5	69.5	136	0.22	0.07
3	10.30	0.50	3	14.5	6.5	410	431	0.95	0.02
Total	20.75		Sum	348.5	40.5	522	911		
			Producer accuracies	0.88	0.65	0.82			
			Confidence interval	0.03	0.15	0.02			

Overall accuracy:	84%
Confidence interval:	2%
Confidence level	95%

Adjusted area

Land use class	Adjusted area, Mha	Error, Mha
1	8.04	0.39
2	0.75	0.22
3	11.96	0.40

Table S7: Confusion matrix for the Caucasus

Land use classes	Mapped area, Mha	Weights	Map/ Validation data set	1	2	3	Sum	User accuracies	Confidence interval
1	4.51	0.17	1	118.5	5.5	51	175	0.68	0.07
2	1.37	0.05	2	28.5	39	51.5	119	0.33	0.09
3	20.56	0.78	3	4	5.5	493.5	503	0.98	0.01
Total	26.43		Sum	151	50	596	797		
			Producer accuracies	0.86	0.55	0.91			
			Confidence interval	0.05	0.16	0.01			

Overall accuracy:	90%
Confidence interval:	2%
Confidence level:	95%

Adjusted area

Land use classes	Adjusted area, Mha	Error, Mha
1	3.55	0.37
2	0.81	0.25
3	22.08	0.41

References

1. Friedl, M. A. *et al.* MODIS Collection 5 global land cover: Algorithm refinements and characterization of new datasets. *Remote Sens. Environ.* **114,** 168–182 (2010).
2. Defourny, P. *et al. Land Cover CCI. Product user guide. V.2*. 87 (UCL-Geomatics, 2014).
3. Fritz, S. *et al.* Mapping global cropland and field size. *Glob. Change Biol.* **21,** 1980–1992 (2015).
4. FAO. Global Land Cover-SHARE (GLC-SHARE). (2015).
5. Jun, C., Ban, Y. & Li, S. China: Open access to Earth land-cover map. *Nature* **514,** 434–434 (2014).
6. Schierhorn, F. *et al.* Post-Soviet cropland abandonment and carbon sequestration in European Russia, Ukraine, and Belarus. *Glob. Biogeochem. Cycles* **27,** 1175–1185 (2013).
7. Prishchepov, A. V., Radeloff, V. C., Baumann, M., Kuemmerle, T. & Müller, D. Effects of institutional changes on land use: agricultural land abandonment during the transition from state-command to market-driven economies in post-Soviet Eastern Europe. *Environ. Res. Lett.* **7,** 024021 (2012).
8. Kraemer, R. *et al.* Long-term agricultural land-cover change and potential for cropland expansion in the former Virgin Lands area of Kazakhstan. *Environ. Res. Lett.* **10,** 054012 (2015).
9. Alcantara, C. *et al.* Mapping the extent of abandoned farmland in Central and Eastern Europe using MODIS time series satellite data. *Environ. Res. Lett.* **8,** 035035 (2013).
10. de Beurs, K. M. & Ioffe, G. Use of Landsat and MODIS data to remotely estimate Russia's sown area. *J. Land Use Sci.* **9,** 377–401 (2013).
11. Schepaschenko, D. *et al.* A new hybrid land cover dataset for Russia: a methodology for integrating statistics, remote sensing and in situ information. *J. Land Use Sci.* **6,** 245–259 (2011).
12. Hansen, M. C. *et al.* High-Resolution Global Maps of 21st-Century Forest Cover Change. *Science* **342,** 850–853 (2013).
13. Estel, S. *et al.* Mapping farmland abandonment and recultivation across Europe using MODIS NDVI time series. *Remote Sens. Environ.* **163,** 312–325 (2015).

14. Bartalev, S. A., Plotnikov, D. E. & Loupian, E. A. Mapping of arable land in Russia using multi-year time series of MODIS data and the LAGMA classification technique. *Remote Sens. Lett.* **7,** 269–278 (2016).

15. Kussul, N. N., Lavreniuk, N. S., Shelestov, A. Y., Yailymov, B. Y. & Butko, I. N. Land Cover Changes Analysis Based on Deep Machine Learning Technique. *J. Autom. Inf. Sci.* **48,** 42–54 (2016).

16. Lavreniuk, M., Kussul, N., Skakun, S., Shelestov, A. & Yailymov, B. Regional retrospective high resolution land cover for Ukraine: Methodology and results. in *2015 IEEE International Geoscience and Remote Sensing Symposium (IGARSS)* 3965–3968 (2015). doi:10.1109/IGARSS.2015.7326693

17. Olofsson, P. *et al.* Good practices for estimating area and assessing accuracy of land change. *Remote Sens. Environ.* **148,** 42–57 (2014).

18. Maus, V., Appel, M. & Giorgino, T. Time-Weighted Dynamic Time Warping for satellite image time series analysis (Software). (2017). Available at: https://cran.r-project.org/web/packages/dtwSat/index.html. (Accessed: 3rd October 2017)
